# Role of the E3 ubiquitin ligase RNF157 as a novel downstream effector linking PI3K and MAPK signaling pathways to the cell cycle

**DOI:** 10.1074/jbc.M117.792754

**Published:** 2017-06-27

**Authors:** Taner Dogan, Florian Gnad, Jocelyn Chan, Lilian Phu, Amy Young, Mark J. Chen, Sophia Doll, Matthew P. Stokes, Marcia Belvin, Lori S. Friedman, Donald S. Kirkpatrick, Klaus P. Hoeflich, Georgia Hatzivassiliou

**Affiliations:** From the Departments of ‡ Translational Oncology,; § Bioinformatics and Computational Biology,; ¶ Microchemistry Proteomics and Lipidomics, and; ** Cancer Immunology, Genentech, Inc., South San Francisco, California 94080 and; ‖ Cell Signaling Technology, Danvers, Massachusetts 01923

**Keywords:** cell cycle, cell signaling, cyclin, E3 ubiquitin ligase, melanoma, CDK2, MAPK, PIK3CA

## Abstract

The interconnected PI3K and MAPK signaling pathways are commonly perturbed in cancer. Dual inhibition of these pathways by the small-molecule PI3K inhibitor pictilisib (GDC-0941) and the MEK inhibitor cobimetinib (GDC-0973) suppresses cell proliferation and induces cell death better than either single agent in several preclinical models. Using mass spectrometry-based phosphoproteomics, we have identified the RING finger E3 ubiquitin ligase RNF157 as a target at the intersection of PI3K and MAPK signaling. We demonstrate that RNF157 phosphorylation downstream of the PI3K and MAPK pathways influences the ubiquitination and stability of RNF157 during the cell cycle in an anaphase-promoting complex/cyclosome–CDH1-dependent manner. Deletion of these phosphorylation-targeted residues on RNF157 disrupts binding to CDH1 and protects RNF157 from ubiquitination and degradation. Expression of the cyclin-dependent kinase 2 (CDK2), itself a downstream target of PI3K/MAPK signaling, leads to increased phosphorylation of RNF157 on the same residues modulated by PI3K and MAPK signaling. Inhibition of PI3K and MEK in combination or of CDK2 by their respective small-molecule inhibitors reduces RNF157 phosphorylation at these residues and attenuates RNF157 interaction with CDH1 and its subsequent degradation. Knockdown of endogenous RNF157 in melanoma cells leads to late S phase and G_2_/M arrest and induces apoptosis, the latter further potentiated by concurrent PI3K/MEK inhibition, consistent with a role for RNF157 in the cell cycle. We propose that RNF157 serves as a novel node integrating oncogenic signaling pathways with the cell cycle machinery and promoting optimal cell cycle progression in transformed cells.

## Introduction

The phosphoinositide 3-kinase (PI3K) and MAPK signaling cascades are involved in a wide variety of biological processes associated with cancer development, such as cell growth, proliferation, and survival. The frequent deregulation of both signaling pathways and the cross-talk between them at multiple points highlight the importance of combinatorial treatment in cancer therapy. Indeed, mounting evidence suggests co-targeting of these pathways is a promising strategy (1, 2). For example, cobimetinib (GDC-0973), a selective inhibitor of MEK, has recently been approved for the treatment of advanced melanoma in combination with the B-RAF inhibitor vemurafenib. Pictilisib (GDC-0941), a potent, selective inhibitor of class I PI3Ks, has demonstrated significant efficacy in both preclinical and clinical studies as a single agent and in combination with other inhibitors (3–5). A subset of cancers displays activation of both the PI3K and MAPK pathways; however, only a relatively small number of substrates at the interface of PI3K and MAPK signaling have been characterized.

Protein phosphorylation is one of the main control mechanisms regulating signal transduction downstream of PI3K and MAPK pathway activation. The global profiling of these networks has not been explored adequately, mainly due to technical challenges. Fortunately, recent advances in mass spectrometry (MS)-based phosphoproteomics provide a high-throughput technology to identify and quantify phosphopeptides in complicated signaling networks (6–8). In this study, we have used label-free MS to investigate the global response of the phosphoproteome to the inhibition of PI3K, MEK, or both by the highly selective, small-molecule inhibitors pictilisib and cobimetinib in melanoma cells. This approach has uncovered the E3 ubiquitin ligase really interesting new gene (RING) finger protein 157 (RNF157)^3^ as a novel, common downstream phosphorylation target of both pathways. Silencing of RNF157 leads to late S and G_2_/M cell cycle arrest, consistent with a role for RNF157 during these cell cycle transitions in cells with PI3K/MAPK activation. Using proteomics, we have identified as putative RNF157-interacting partners several mitochondrial ribosomal and RNA-binding proteins. We demonstrate that cell cycle regulators CDK2 and CDH1 can both bind to RNF157 and regulate it in a cell cycle-dependent manner. CDK2 promotes phosphorylation of RNF157 at the same Ser^660–663^ residues that become phosphorylated downstream of combined PI3K and MAPK pathway activity. Phosphorylation at these same residues on RNF157 plays a promoting role for RNF157 recognition by CDH1 and its subsequent proteasomal degradation during late mitosis and early G_1_. These findings reveal a novel cross-talk mechanism between oncogenic signaling pathways and cell cycle components through the RING finger E3 ubiquitin ligase RNF157.

## Results

### Quantitative phosphoproteomics identifies the E3 ubiquitin ligase RNF157 as a downstream effector of PI3K and MAPK signaling

To identify downstream effectors of the PI3K and MAPK pathways, we used the BRAF^V600E^ mutant human melanoma cell lines A2058 and 624MEL in which both pathways are activated, and thus the combination of PI3K and MEK inhibitors maximally reduces their activity and induces cell death (supplemental Fig. S1). We measured differences in phosphorylation upon treatment of these cell lines with the PI3K inhibitor pictilisib (GDC-0941), the MEK inhibitor cobimetinib (GDC-0973), or the combination of both compounds. To this end, we used the label-free based PTMScan® method from Cell Signaling Technology (9, 10). Western blot screening of the protein lysates using AKT and MAPK phosphomotif antibodies showed evident global phosphorylation changes after treatments and confirmed the decrease of markers for PI3K or MAPK activity, including phospho-AKT, phospho-ERK1/2, and phospho-S6 (supplemental Fig. S1C). Following phosphopeptide enrichment with the MAPK and AKT phosphomotif antibodies, we applied mass spectrometric analysis as described previously (10) in the A2058 cell line (supplemental Fig. S2A) and identified 1135 phosphorylation sites from 603 proteins (supplemental Fig. S2B and supplemental Table S1).

**Figure 1.**
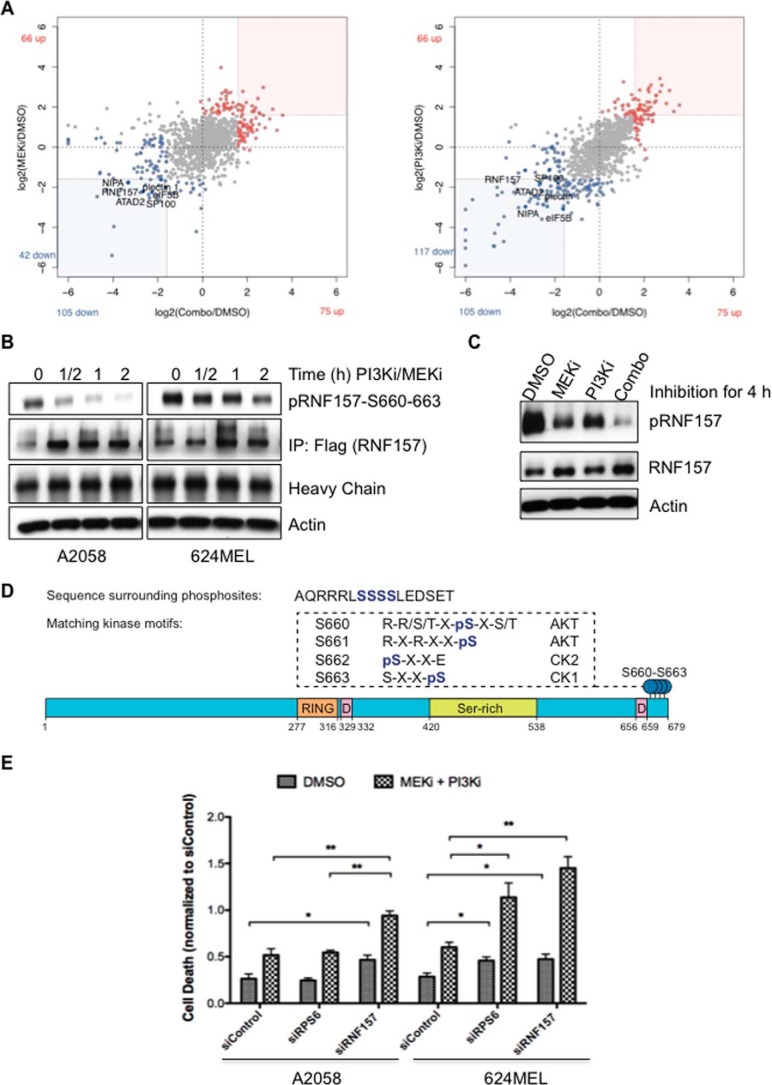
**Phosphoproteomic identification of PI3K/MAPK pathway nodes.** *A*, scatter plots illustrate peptide phosphorylation changes after single inhibition of MAPK or dual inhibition (*Combo*) of MAPK and PI3K pathways in A2058 cells. *B* and *C*, Western blotting confirmed the decreased phosphorylation of immunoprecipitated FLAG-tagged RNF157 (*B*) and endogenous RNF157 (*C*) in response to dual (0.25 μm each for the times indicated (*B*)) and single inhibition (0.5 μm each for 2 h) with the phosphospecific RNF157 antibody. *D*, schematic diagram of protein domains of RNF157 and some key facts. RING domain contains a Cys_3_-His-Cys_4_ motif that binds two zinc cations. Most of the RING domain-containing proteins function as ligases. The D-boxes are consensus sequences for targeted polyubiquitination. The phosphorylation sites identified by MS-based phosphoproteomics are located at the C terminus of RNF157 and represent the phosphorylation motifs for AKTs and casein kinases (*CK*). *E*, inhibition of RNF157 enhances cellular efficacy by PI3K inhibitor (*PI3Ki*) pictilisib (GDC-0941) and MEK inhibitor (*MEKi*) cobimetinib (GDC-0973). A2058 and 624MEL cells were treated with siRNA against RNF157 or RPS6 or transfected with non-targeting siRNA as a control for 48 h and subsequently treated with GDC-0973 and GDC-0941 (0.03125 μm each (A2058 cells) or 0.0625 μm each (624MEL cells)) for 24 h. Cell death was evaluated by quantifying BrdU incorporation and cytoplasmic histone-associated DNA fragments, respectively. Data from three independent experiments are shown. *Error bars* represent ±S.D. of the mean. A *p* value of <0.05 was considered statistically significant. *p* values are designated with *asterisks* as follows: *, *p* ≤ 0.05; **, *p* ≤ 0.01. *IP*, immunoprecipitation.

After inhibition of both PI3K and MEK, 75 of 1045 (7%) and 105 of 1045 (10%) phosphopeptides showed up- or down-regulation, respectively (Fig. 1*A*). To characterize responding phosphoproteins, we applied gene set enrichment analysis using collections from the Molecular Signatures Database (11). We found that many proteins that showed increased phosphorylation after PI3K, MEK, or combination treatment were associated with the cytoskeleton (*p* < 0.01) (supplemental Table S2). Proteins with decreased phosphorylation after treatments were commonly involved in the cell cycle (*p* < 0.01), including CDK2, CDC2, and TOP2A.

Although there was a significant enrichment of phosphoproteins with known biological roles, we were more interested in phosphoproteins that had not been previously associated with PI3K or MAPK signaling. The E3 ubiquitin ligase RNF157 showed substantial down-regulation of phosphorylation after MEK or PI3K inhibition and maximal blockade of phosphorylation after dual inhibition in both melanoma cell lines (Fig. 1, *A–C*, and supplemental Table S1). Because RNF157 is a relatively understudied E3 ubiquitin ligase belonging to the RING finger family and represents a novel effector of the PI3K and MAPK pathways, we focused on further characterizing its role. We detected several distinct RNF157 phosphopeptides by MS showing single, double, triple, or quadruple phosphorylation in the Ser^660–663^ region (supplemental Table S3). Western blotting with a novel phosphospecific RNF157 antibody that we generated, as described under “Materials and methods,” and validated for its specificity against pRNF157^S660–663^ (supplemental Fig. S2C) confirmed a rapid, time-dependent decrease in RNF157 phosphorylation after combination treatment (Fig. 1, *B* and *C*). Sequence analysis of RNF157 revealed that the identified phosphorylation sites Ser^660–663^ were localized adjacent to one of two putative destruction box (D-box) motifs composed of the sequence R*XX*L*XXXX*N (Fig. 1*D*). D-box-containing proteins play key roles in cell cycle regulation and are targeted for degradation by the APC/C E3 ligase complex. We performed siRNA knockdown of RNF157 in the presence or absence of PI3K and MEK inhibitors to evaluate the role of RNF157 in tumor cell survival and found that, whereas RNF157 knockdown modestly increased cell death compared with siControl, simultaneous treatment with siRNF157 and PI3K/MEK inhibitors led to significantly greater cell death than inhibitor treatment alone (Fig. 1*E*).

RNF157 is paralogous to the E3 ubiquitin ligase MGRN1 with 42.8% sequence identity, mainly in the N terminus (supplemental Fig. S3A). MGRN1 has been implicated in endosomal trafficking, prion turnover, and melanocortin-2 receptor stability (12–15). RNF157 and MGRN1 are both conserved in jawed vertebrates (supplemental Fig. S3B) and have a single co-ortholog in most eukaryotes other than fungi. Their co-ortholog in *Drosophila*, CG9941, interacts with the protein CG5334, whose human homolog, MKRN1, has been reported as an E3 ligase for p53 and p21 and implicated in cell cycle regulation (16). Importantly, the C-terminal tail of human RNF157, containing the Ser^660–663^ phosphorylation cluster we identified, is found only in eutherian mammals and not in MGRN1 (supplemental Fig. S3, A and C), suggesting a potentially unique role for this phosphorylation cluster region in mammalian RNF157. According to the secondary structure prediction programs JPred4 (17) and RONN (18), the Ser^660–663^ region is predicted to be a highly solvent-exposed, hydrophilic, and disordered region of RNF157 with no defined secondary structure (supplemental Fig. S3D).

### Cell cycle-dependent regulation of RNF157 expression

**Figure 2.**
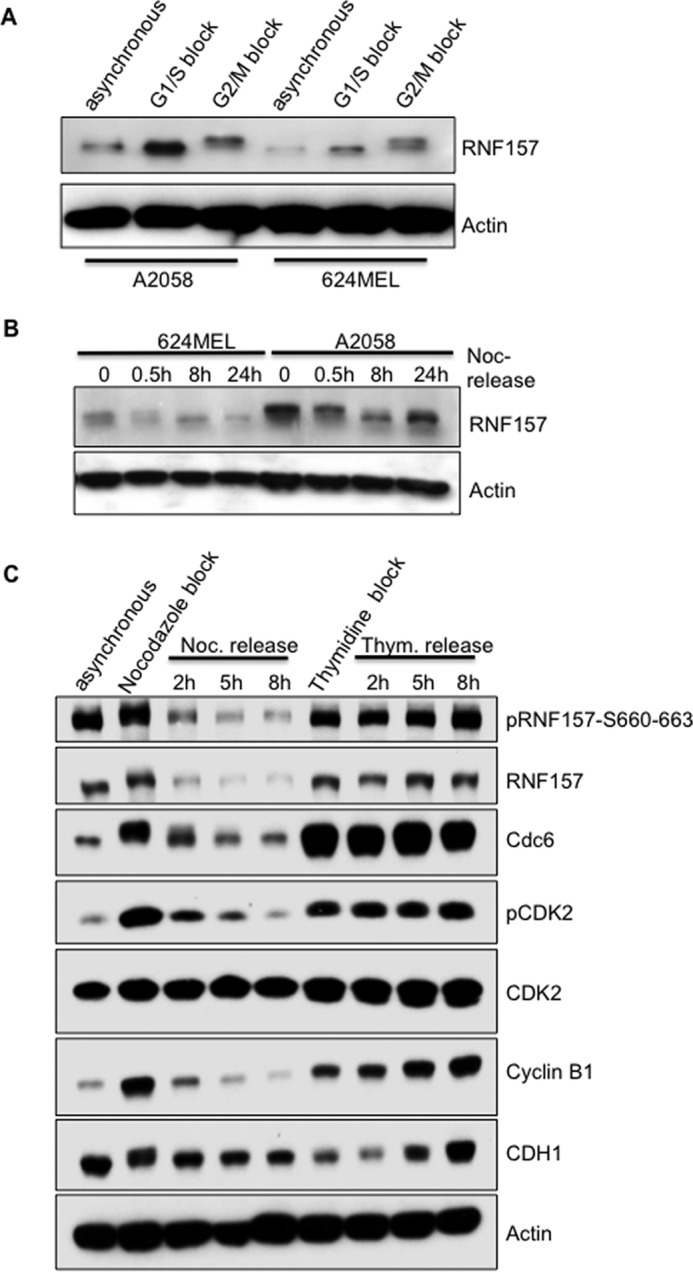
**RNF157 fluctuates in a cell cycle-dependent manner.** *A*, A2058 and 624MEL melanoma cells were arrested at the G_1_/S boundary with a double thymidine treatment and at the G_2_/M boundary with a nocodazole treatment. Blots of the cell lysates were probed for endogenous RNF157 and actin as a loading control. *B*, A2058 and 624MEL melanoma cells were arrested in mitosis with thymidine (*Thym*.)/nocodazole (*Noc.*) treatment and then released into fresh medium. Cells were collected for the times indicated and analyzed by Western blotting for endogenous RNF157 expression. *C*, A2058 cells were synchronized as in *A*, released into fresh medium, collected for the times indicated, and analyzed by Western blotting with the indicated antibodies. *pCDK2* represents the Thr(P)^160^ site.

It has been reported that RNF157 is predominantly expressed in the brain (19), but there are no reports of RNF157 expression levels in proliferating cells. Given that RNF157 contains D-box motifs characteristic of APC/C–CDH1-regulated proteins and has a distant connection to MKRN1, which is involved in cell cycle control, we wanted to evaluate the role of RNF157 in the cell cycle. We first assessed RNF157 expression via immunoblotting during cell cycle progression and observed that RNF157 levels and molecular weight fluctuate in a cell cycle-dependent manner in both A2058 and 624MEL melanoma cells (Fig. 2, *A–C*). Compared with unsynchronized cells, RNF157 protein levels were higher in cells arrested in G_1_/S and G_2_/M (Fig. 2*A*). The apparent molecular weight of RNF157 was increased in nocodazole-synchronized G_2_/M cells and gradually decreased as the arrested cells were released into fresh media (Fig. 2*B*). In contrast, the RNF157 migration pattern remained constant in nocodazole-synchronized cells treated with the proteasome inhibitor MG132 at the time of the release into fresh media (supplemental Fig. S4A). The fluctuation of RNF157 levels and migration pattern mirror those of the cell cycle regulators cyclin B1 and CDC6 during the cell cycle (Fig. 2*C*). Both cyclin B1 and CDC6 are substrates of the APC/C–CDH1 E3 ligase complex and are subject to phosphorylation by cyclin-dependent kinases, including CDK2 (20, 21). Interestingly, CDK2 activation, as measured by blotting with a CDK2 Thr(P)^160^ antibody, was modulated with kinetics that matched RNF157 phosphorylation and protein levels (Fig. 2*C*). These results prompted us to hypothesize that RNF157 regulation during the cell cycle may be linked to the APC/C–CDH1 complex and to CDK2.

### Role of CDH1 in RNF157 stability

**Figure 3.**
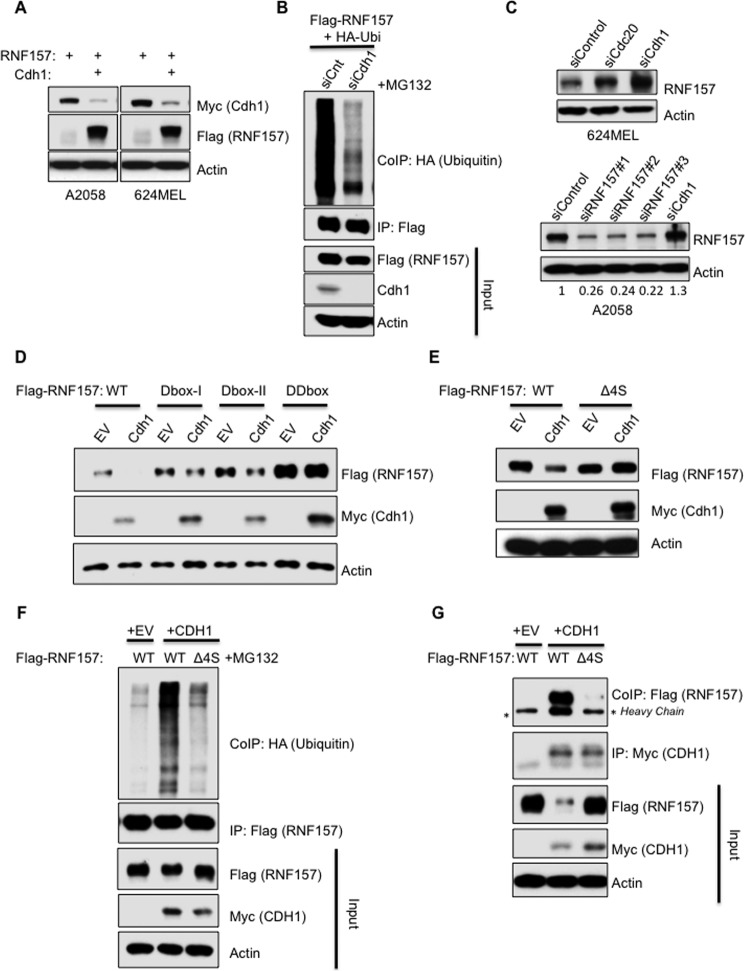
**CDH1 is involved in the regulation of RNF157 degradation.** *A*, A2058 and 624MEL cells were transfected with FLAG-RNF157 together with control vector or Myc-CDH1 expression vector. The cell lysates were then immunoblotted with FLAG, Myc, and actin antibodies. The latter served as a loading control. *B*, HeLa cells were transfected with control siRNA (*siCnt*) or CDH1 siRNA (*siCdh1*) for 24 h, then transfected with FLAG-RNF157 and HA-ubiquitin expression vectors for an additional 24 h, and treated with 10 μm MG132 for 4 h prior to harvest. Ubiquitination of immunoprecipitated FLAG-RNF157 was identified by HA antibody against HA-tagged ubiquitin co-immunoprecipitated with FLAG-RNF157. *C*, 624MEL and A2058 cells were transfected with control siRNA (*siControl*) and siRNAs against CDH1 (*siCdh1*), CDC20 (*siCdc20*), and RNF157 (*siRNF157#1–3*) separately for 48 h. Lysates were then analyzed by Western blotting to detect the endogenous levels of RNF157 using the total RNF157 antibody. Actin served as a loading control. Band intensities were quantified with ImageJ. The ratio of RNF157 compared with actin was determined and expressed as a proportion of the control siRNA sample. *D*, A2058 cells were transfected with wild-type FLAG-RNF157 (*WT*), D-box I mutant (*Dbox-I*), D-box II mutant (*Dbox-II*), and double D-box mutant (*DDbox*) together with control vector (*EV*) or Myc-CDH1. Cells were lysed 48 h post-transfection and analyzed by Western blotting using the indicated antibodies. *E*, lysates of A2058 cells transfected with wild-type RNF157 or phosphodeficient mutant (Δ4S) together with control vector (*EV*) or Myc-tagged CDH1 expression plasmid were subjected to immunoblotting with the antibodies as indicated. *F*, analysis of ubiquitination of immunoprecipitated FLAG-RNF157 and phosphodeficient RNF157 mutant (Δ4S) co-transfected with Myc-CDH1 and HA-ubiquitin in HeLa cells treated with 10 μm MG132 for 4 h prior to harvest. Immunoprecipitation performed with FLAG antibody. Co-immunoprecipitated ubiquitin was detected with HA antibody. *G*, Western blot analysis of FLAG-RNF157 and FLAG-tagged phosphodeficient RNF157 mutant (Δ4S) co-immunoprecipitated with Myc-CDH1 from HeLa cells transfected with the indicated plasmids. *IP*, immunoprecipitation, *CoIP*, co-immunoprecipitation.

As mentioned above, sequence analysis of RNF157 revealed that it contains two putative D-box motifs, one of which is localized adjacent to the identified phosphorylation sites Ser^660–663^ (Fig. 1*D*). During the cell cycle, D-box-containing proteins are regulated by the APC/C ubiquitin ligase complex via either of two substrate-recruiting proteins, CDH1 or CDC20 (22). We tested whether CDH1 or CDC20 overexpression can modulate RNF157 levels and found that overexpression of CDH1 led to a decrease in RNF157 protein levels in both A2058 and 624MEL cell lines (Fig. 3*A*). CDH1 overexpression in this experiment did not appear to have general effects on other cell cycle regulators, like cyclin B1, itself a APC/C–CDH1 target (supplemental Fig. S4C). Silencing of CDH1 by siRNA reduced the high levels of RNF157 ubiquitination observed in the presence of the proteasome inhibitor MG132 (Fig. 3*B*) and led to increased levels of endogenous RNF157 *versus* modest effects upon silencing of CDC20 (Fig. 3*C*). In addition, CDC20 overexpression did not affect RNF157 levels (supplemental Fig. S4B). These data support the model that CDH1, rather than CDC20, can modulate the ubiquitination and stability of RNF157, consistent with RNF157 being a putative substrate of the APC/C–CDH1 E3 ligase complex.

To identify the structural elements regulating RNF157 stability, we mutated the two canonical D-box motifs on RNF157 individually and in combination. Mutation of either D-box motif partially stabilized RNF157, whereas simultaneously mutating both D-boxes maximally enhanced RNF157 stability, making it refractory to CDH1 overexpression (Fig. 3*D*). Notably, D-box deficiency did not impair the interaction between RNF157 and CDH1 (supplemental Fig. S4D).

Phosphorylation of cell cycle proteins often regulates their degradation and modulates their interactions with other cell cycle components (23–25). Therefore, we explored the impact of RNF157 phosphorylation at Ser^660–663^ on its regulation by CDH1. We generated a phosphodeficient mutant version of RNF157 where the Ser^660–663^ serine cluster was deleted (Δ4S). In contrast to wild-type RNF157, the Δ4S mutant was stable in the presence of overexpressed CDH1 (Fig. 3*E*). Furthermore, the Δ4S mutant displayed significantly reduced ubiquitination following CDH1 overexpression (Fig. 3*F*) and lost the ability to interact with CDH1 (Fig. 3*G*). Given that these residues are predicted to lie in a disordered region (supplemental Fig. S3D), it is unlikely that their deletion would introduce a structural change that could account for these effects independently of the Ser^660–663^ cluster. Taken together, these data suggest that the Ser^660–663^ residues of RNF157 promote APC/C–CDH1-mediated RNF157 ubiquitination and proteasomal degradation by promoting APC/C–CDH1/RNF157 binding, whereas RNF157 D-box motifs are dispensable for RNF157 binding to APC/C–CDH1 but are required for RNF157 degradation.

### RNF157 regulation by PI3K, MEK, and CDK2 activity

**Figure 4.**
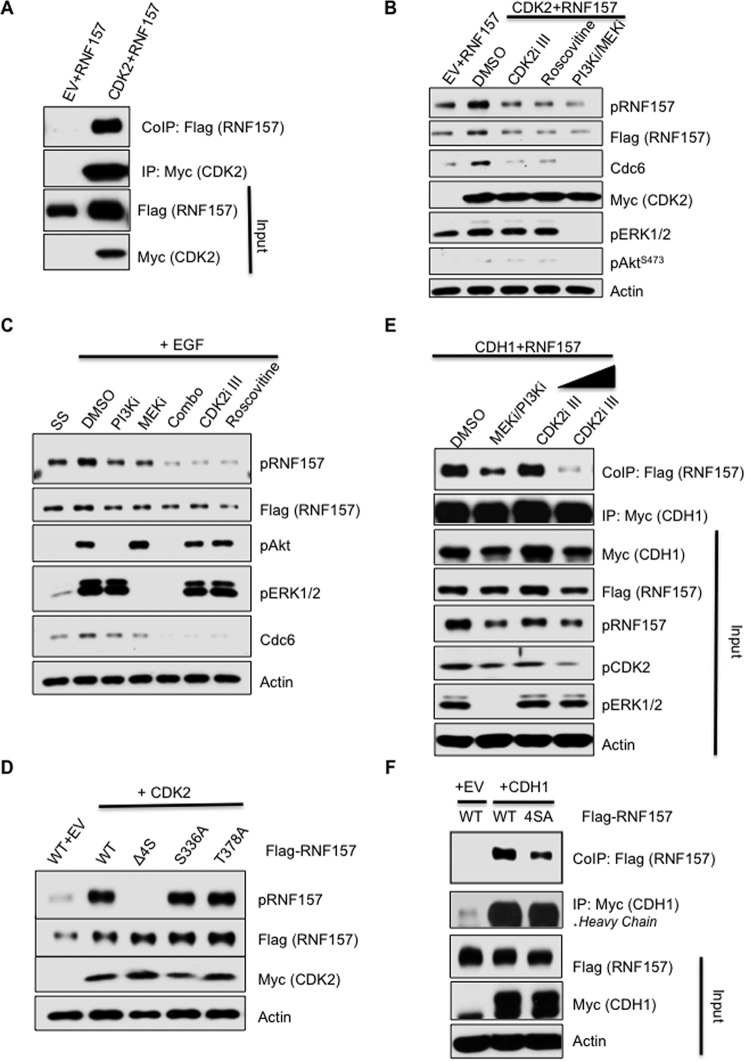
**CDK2 promotes RNF157 phosphorylation.** *A*, Western blot of FLAG-RNF157 co-immunoprecipitated with Myc-CDK2 from 624MEL melanoma cells transfected with the indicated vector combinations. *B*, analysis of the phosphorylation levels of FLAG-RNF157 by using the phosphospecific RNF157 antibody (pRNF157^S660–663^) upon treatment with different inhibitors. RNF157 was co-transfected with control vector or Myc-CDK2 and where indicated was treated with DMSO as a control, CDK2 inhibitors CDK2i III (4.2 μm) and roscovitine (20 μm), or PI3K inhibitor/MEK inhibitor (*PI3Ki/MEKi*) in combination (*Combo*) for 6 h. *C*, HeLa cells were transfected with FLAG-RNF157 and serum-starved at 24 h post-transfection for 16 h. The cells were then treated with the inhibitors as indicated and lysed after EGF treatment (100 ng/ml for 5 min). *SS lane*, serum-starved unstimulated cells. Phosphorylation of RNF157 was analyzed by immunoblotting with the phosphospecific antibody. *D*, lysates of HeLa cells transfected with wild-type RNF157 or different phosphomutant plasmids together with control vector (*EV*) or Myc-CDK2 vector. Immunoblotting was performed with pRNF157^S660–663^, FLAG, Myc, and actin antibodies. *E*, lysates of HeLa cells transfected with FLAG-RNF157 and Myc-CDH1 expression plasmids and treated with the indicated inhibitors were subjected to immunoprecipitation with the Myc antibody followed by immunoblotting with FLAG, Myc, pRNF157^S660–663^, pRNF157^T170^, phospho-ERK (*pERK*), and actin antibodies. CDK2 inhibitor was used in two different concentrations, 2.1 and 4.2 μm, for 8 h. *F*, lysates of HeLa cells transfected with control vector (*EV*), FLAG-RNF157, FLAG-RNF157(4SA), and Myc-CDH1 expression plasmids were subjected to immunoprecipitation with the Myc antibody followed by immunoblotting with FLAG, Myc, and actin antibodies. *IP*, immunoprecipitation, *CoIP*, co-immunoprecipitation; *pAkt*, phospho-AKT; *pERK1/2*, phospho-ERK1/2; *pCDK2*, phospho-CDK2.

Previous studies have implicated PI3K in cell cycle control through CDK2 (26–29). Because RNF157 phosphorylation coincides with CDK2 activation and downstream target regulation (Fig. 2*C*), we asked whether CDK2 kinase activity could modulate RNF157 Ser^660–663^ phosphorylation downstream of PI3K/MAPK signaling. First, we assessed the interaction between CDK2 and RNF157 in 624MEL cells overexpressing both proteins (Fig. 4*A*). Next, we assessed the effect of overexpressed CDK2 on the phosphorylation status of Ser^660–663^ in cells treated either with DMSO, two different CDK2 inhibitors, or a PI3K/MEK inhibitor combination. Compared with vector control, we detected increased phosphorylation of RNF157 upon CDK2 overexpression that was abolished by treatment with either of the CDK2 inhibitors or the PI3K/MEK inhibitor combination (Fig. 4*B*). This is consistent with previous reports that CDK2 activity is activated downstream of PI3K/MAPK signaling (26–29). Treatment with these inhibitors, however, also led to an apparent decrease in the total levels of RNF157, mirroring effects on CDC6, a CDK2 target and APC/C–CDH1 substrate whose stability depends on CDK2 phosphorylation (20). This may be due to the release of the negative regulation of APC/C–CDH1 by CDK2 (30) during the 6 h of inhibitor treatment. To test the effects of PI3K/MEK and CDK2 inhibitors on Ser^660–663^ phosphorylation in an acute system to prevent effects on total RNF157 levels, we assessed pRNF157^S660–663^ levels 5 min after epidermal growth factor stimulation in the absence *versus* presence of inhibitors. Acute EGF stimulation induced a rapid increase in pRNF157^S660–663^ levels, concomitant with an increase in total levels of the CDK2 substrate CDC6, whose stability is positively regulated by CDK2 phosphorylation (20) (Fig. 4*C*). Treatment with either the PI3K/MEK inhibitor combination or with two different CDK2 inhibitors abrogated pRNF157^S660–663^ levels in the absence of any change in total RNF157 levels and concomitant with a decrease in CDC6 levels as a readout for suppression of CDK2 activity (Fig. 4*C*). Deletion of Ser^660–663^ residues (Δ4S) abolished CDK2-induced RNF157 phosphorylation (Fig. 4*D*). These data, along with the earlier observation that CDK2 inhibition blocked pRNF157^S660–663^ levels upon growth factor signaling, are consistent with the conclusion that the Ser^660–663^ cluster that we identified as being co-regulated by the PI3K/MAPK pathways is phosphorylated downstream of CDK2 activation induced by PI3K/MEK signaling. RNF157 does not contain a consensus CDK phosphorylation motif ((S/T)P*X*(K/R)); therefore, the kinase phosphorylating Ser^660–663^ is likely a CDK2-regulated kinase rather than CDK2 itself. Interestingly, RNF157 contains a Cy (R*X*L) cyclin-binding motif immediately preceding Ser^660^ and within D-box II (Cy = ^657^RRL^659^) (31). This Cy motif is embedded within the R*XX*S consensus AKT motif ^657^RRLS^660^ immediately preceding the Ser^660–663^ cluster. The presence of a Cy motif has been shown to increase the affinity of an SP*X*K-containing peptide for CDK2 75–120-fold (31) and may facilitate CDK2 interaction with RNF157.

Having previously shown that the Ser^660–663^ cluster is required for CDH1–RNF157 complex formation and ubiquitination (Fig. 3, *F* and *G*), we tested the consequences of Ser^660–663^ cluster phosphorylation on the CDH1/RNF157 interaction using (*a*) PI3K/MEK and CDK2 inhibitors and wild-type FLAG-RNF157 and (*b*) a serine 660–663-to-alanine (4SA) mutant FLAG-RNF157 to block Ser^660–663^ phosphorylation. We found that inhibitor treatments led to decreased CDH1/RNF157 interaction, proportionally to the accompanying decreases in pRNF157^S660–663^ and pCDK2^T160^ levels; the latter is used as a readout of CDK2 activation (Fig. 4*E*). Serine-to-alanine substitutions of the Ser^660–663^ cluster led to decreased CDH1/RNF157 interaction compared with WT RNF157 (Fig. 4*F*), consistent with the decrease in CDH1/RNF157 binding observed after MEK/PI3K inhibitor treatment (Fig. 4*E*). Altogether, these data support a model in which RNF157 phosphorylation at the Ser^660–663^ cluster occurs downstream of CDK2 activation, induced by PI3K/MEK (26–29), and promotes RNF157/CDH1 interaction. Because some degree of RNF157/CDH1 interaction remains even when Ser^660–663^ phosphorylation is blocked by kinase inhibitors or Ser-to-Ala point mutations, we conclude that phosphorylation of the Ser^660–663^ cluster enhances the CDH1/RNF157 interaction but is not absolutely required.

### Both CDH1 and CDK2 are involved in the modulation of RNF157 stability during the cell cycle

**Figure 5.**
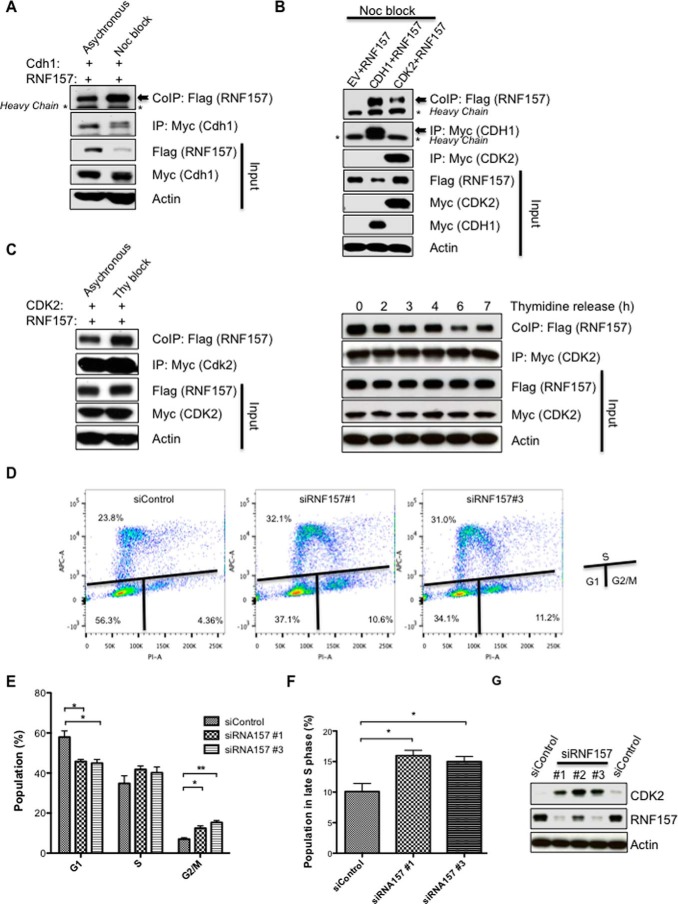
**RNF157 role within the cell cycle.** *A*, FLAG-RNF157 was co-transfected with Myc-CDH1 in HeLa cells and co-immunoprecipitated with Myc-CDH1 after DMSO or nocodazole (*Noc*) treatment. Lysates were subjected to immunoblotting using FLAG, Myc, and actin antibodies. *B*, Western blotting of RNF157 co-immunoprecipitated with CDH1 or CDK2 from HeLa cells arrested in G_2_/M by nocodazole. *C*, FLAG-RNF157 co-immunoprecipitated with Myc-CDK2 from HeLa cells with/without double thymidine (*Thy*) block. Western blot analysis was performed as in *A*. Cells were transfected and arrested with thymidine as in the *left panel* and then released into fresh medium for the times indicated. Western blots of FLAG-RNF157 co-immunoprecipitated with Myc-CDK2 were analyzed with the antibodies as indicated. *D*, analysis of cell cycle progression of HeLa cells transfected with control siRNA or two different siRNAs against RNF157. Samples were stained with the Click-iT EdU Assay kit using the manufacturer's recommended protocols 4 days post-transfection, and their DNA content was determined by flow cytometry. The plots show cells in different cell cycle phases. Cell cycle analysis was performed by gating G_1_, S, and G_2_/M on propidium iodide (*PI-A*) for DNA content and on Alexa Fluor 647 (*APC-A*) for EdU incorporation. *E* and *F*, quantification of the cell cycle analysis represented in *D*. Data represent means ± S.D. (*error bars*) obtained from three independent experiments to indicate a statistically significant result. *p* values are designated with *asterisks* as follows: *, *p* ≤ 0.05; **, *p* ≤ 0.01. *G*, HeLa cells were transfected with control siRNA or three different siRNAs against RNF157 for 4 days. The lysates were then subjected to immunoblotting to detect the endogenous proteins of interest by using the antibodies indicated. *IP*, immunoprecipitation, *CoIP*, co-immunoprecipitation.

Phosphorylation of CDH1 by CDK2 blocks the binding of CDH1 to the APC/C ubiquitin ligase complex and inactivates this complex during late G_1_ and S phases (30). We thus investigated the binding pattern of RNF157 to both cell cycle modulators during the cell cycle. The interaction between RNF157 and CDH1 was higher in cells arrested at G_2_/M compared with unsynchronized cells (Fig. 5*A*), and both CDH1 and CDK2 could bind to RNF157 during G_2_/M (Fig. 5*B*). The binding of RNF157 to CDK2 was higher in cells arrested during G_1_/S compared with unsynchronized cells (Fig. 5*C*, *left panel*) and gradually decreased after cells were released into fresh media post-thymidine block (Fig. 5*C*, *right panel*). We previously showed that optimal binding of RNF157 to CDH1 requires Ser^660–663^ phosphorylation downstream of PI3K/MEK and CDK2 activity (Figs. 3 and 4*E*). However, even though RNF157 may bind to CDH1 soon after it becomes phosphorylated at Ser^660–663^ early on in the cell cycle and maintains high CDH1 binding during the G_2_/M transition, RNF157 levels appear to decrease gradually following release of melanoma cells from nocodazole-induced G_2_/M arrest into mitosis and G_1_, consistent with its degradation occurring during late mitosis and early G_1_ (Fig. 2*C* and supplemental Fig. S5). This timeline matches the reported inhibition of CDH1 activity by CDK2, occurring from G_1_/S until late M phase at which point CDH1 becomes active and stays active during G_1_ (30). Thus, we propose that CDK2 may help coordinate RNF157 stability with the cell cycle by maintaining the APC/C–CDH1 complex inactive during G_1_/S, S, and G_2_/M while at the same time promoting CDH1/RNF157 interaction via RNF157 Ser^660–663^ phosphorylation. As a result, RNF157 remains stable from G_1_/S until G_2_/M and able to play its role in the cell cycle but is primed to be rapidly degraded as soon as the APC/C–CDH1 complex becomes active in late M (supplemental Fig. S5).

To evaluate the role of RNF157 in the cell cycle, we next investigated the effects of RNF157 depletion on cell cycle progression. Silencing of endogenous RNF157 by two independent siRNAs increased the number of cells arrested in late S phase as well as the number of cells with a 4N DNA content, indicative of a G_2_/M arrest (Fig. 5, *D–F*). Interestingly, RNF157-depleted cell lysates showed an increase in CDK2 levels, suggesting a potentially reciprocal role of RNF157 in regulating CDK2 (Fig. 5*G*). This observation warrants further investigation, including the assessment of CDK2 as a potential ubiquitination target downstream of RNF157 activity.

The phenotype of RNF157 knockdown cells is consistent with a potential role for RNF157 during late S and G_2_/M transition. To gain a better understanding of the cellular role of RNF157, we undertook a proteomic approach to search for RNF157-interacting proteins in melanoma cells overexpressing FLAG-tagged GFP *versus* FLAG-tagged RNF157. As shown in supplemental Table S4, several proteins were pulled down specifically with immunoprecipitated RNF157-FLAG but not GFP-FLAG from two independent melanoma lines. Interestingly, many of these putative RNF157-interacting proteins are implicated in RNA processing and translation, including several mitochondrial ribosomal proteins (RM19, RT18B, and RT02). Mitochondrial ribosomal proteins are synthesized during G_1_/S, peak in abundance during S phase, subsequently get degraded during M phase (32), and therefore are expressed in the same cell cycle window as RNF157. Further validation of these putative interactive partners and the role of RNF157 in their regulation in future studies may shed light into the mechanistic role of RNF157 during cell cycle progression.

## Discussion

The PI3K and MAPK pathways intersect at multiple levels (33, 34), and combined inhibition of these pathways in tumors shows a stronger effect on apoptosis induction and growth inhibition than individual pathway inhibition (3, 5). One of the key integration points between the PI3K and MAPK pathways is the cell cycle machinery, itself an attractive domain for identifying novel diagnostic and therapeutic targets. Both PI3K and MAPK signaling pathways have been reported to regulate the activation of CDK2, which plays a key role in cell cycle progression, including the regulation of the APC/C–CDH1 E3 ligase complex (26–30). Our study reveals that RNF157, a novel E3 ubiquitin ligase, acts at the interface between the PI3K and MAPK pathways and the cell cycle machinery to promote cell cycle progression and tumor cell survival.

Proper regulation of protein ubiquitination and degradation by the APC and SCF (skp1–cul1–F-box-protein) ubiquitin ligase complexes are key to maintaining the integrity of the cell cycle. Although the SCF ligases target substrates with F-box degrons during the G_1_/S, S, and G_2_ phases, APC ligases are mainly active during M phase and are required to drive progression and exit from mitosis by inducing the proteolysis of key cell cycle regulators through the recognition of D-box or KEN box motifs by the CDC20 and CDH1 APC adaptor proteins (35–40). Our work introduces the D-box-containing protein RNF157 as a candidate mitotic APC/C–CDH1 substrate. In support of RNF157 being a putative APC/C–CDH1 target, two of the four D-box motifs on RNF157 show strong correlations with the consensus D-box motif (R*XX*L*XXXX*N) recognized by the APC/C–CDH1 E3 ligase complex, and unlike wild-type RNF157, RNF157 mutants lacking one or both of these D-box motifs are stable in the presence of overexpressed CDH1 (Fig. 3*D*). In addition, siRNA-mediated silencing of endogenous CDH1 leads to a decrease in RNF157 ubiquitination (Fig. 3*B*) and to an increase in endogenous RNF157 protein levels (Fig. 3*C*). To definitively establish RNF157 as a direct APC/C–CDH1 target, additional *in vitro* experiments using recombinant RNF157 and reconstituted APC/C–CDH1 complex are needed; however, such experiments are complicated by the fact that sufficient quantities of purified APC/C–CDH1 are difficult to obtain (41).

Protein phosphorylation is a key control mechanism regulating signaling pathways downstream of PI3K and MAPK activation. In addition to phosphorylation, there is precedence for extensive cross-talk between phosphorylation and ubiquitination as part of the regulation of cell cycle progression (42). In this study, we have identified the RNF157 phosphosites Ser^660–663^as playing a key role in the cell cycle-dependent regulation of RNF157 stability. This does not exclude the possibility that additional phosphorylation sites of RNF157 (43–45) may also contribute to its regulation. However, it appears that the Ser^660–663^ region is key for regulating RNF157 interactions with the CDH1 component of the APC/C complex and thus RNF157 stability. RNF157 phosphorylation at Ser^660–663^ oscillates during the cell cycle in concert with CDK2 activation and downstream target CDC6 (Figs. 2*C* and 4). Furthermore, CDK2 inhibition blocks growth factor-stimulated increase in pRNF157^S660–663^ to the same degree as PI3K/MEK inhibition (Fig. 4*C*), supporting the model that CDK2 and the kinases it regulates act downstream of PI3K/MEK in a linear pathway to regulate RNF157 stability in concert with its other substrates and with the APC/C–CDH1 complex itself. Preliminary data showing endogenous CDK2 levels increasing in RNF157 knockdown cells (Fig. 5*G*) may point to a reciprocal role for RNF157 in modulating CDK2 levels, an area that warrants further investigation.

Given the scarcity of robust detection reagents for studying endogenous RNF157 levels as they are dynamically regulated and interact with the cell cycle machinery, we have had to rely on exogenous RNF157 for most of our mechanistic studies. Additional studies are needed to address the role of endogenous RNF157 and its regulation and role in the cell cycle. Despite these limitations, we have demonstrated that knockdown of endogenous RNF157 leads to cell cycle arrest during the late S phase and G_2_/M checkpoint in tumor cells (Fig. 5, *D–F*), supporting a role of RNF157 in promoting cell cycle progression. In addition, we have shown that endogenous RNF157 knockdown increases apoptosis in combination with PI3K/MAPK pathway inhibition in melanoma cells (Fig. 1*E*). In an effort to further characterize the role of RNF157, we have identified putative RNF157-interacting proteins by mass spectrometry-based proteomics, including several proteins involved in RNA processing and ribosome biogenesis (supplemental Table S4).

In conclusion, our data support a model in which phosphorylation of RNF157 at Ser^660–663^ downstream of PI3K/MEK/CDK2 activity promotes RNF157 interaction with CDH1. This interaction, however, does not lead to RNF157 degradation until the APC/C–CDH1 complex, normally under negative CDK2 control, becomes active during late mitosis and G_1_ (supplemental Fig. S5). We propose that such coordinated and controlled degradation of RNF157 promotes proper exit from mitosis and cell cycle progression. The fact that knockdown of RNF157 in asynchronous cells has negative consequences for the cell cycle suggests that RNF157 plays an important role during the early phases of the cell cycle. Although the precise function of RNF157 remains unclear, our data suggest that inhibition of RNF157 in combination with PI3K/MAPK inhibition may provide therapeutic benefit to patients whose tumors show coordinate activation of the PI3K and MAPK pathways.

## Materials and methods

### Antibodies

Total RNF157 and RNF157 Ser(P)^660–663^ antibodies were generated by YenZyme Antibodies, LLC. For the phosphospecific RNF157 antibody generation, rabbits were immunized with the phosphopeptide (CRNAQRRRLpSpSpSpSLED-amide where pS is phosphorylated serine) corresponding to residues 652–666 of RNF157. The phosphospecific antibody was then purified in two steps: 1) positive selection using the same phosphopeptide followed by 2) negative selection against the non-phosphopeptide to remove any cross-reactive antibody components. Antibodies against CDK2 (78B2), CDK2 Thr(P)^160^, CDC6 (C42F7), cyclin B1, Myc tag (9B11), AKT, AKT Ser(P)^473^, ubiquitin (P4D1), and HA tag (C29F4) were obtained from Cell Signaling Technology. Anti-actin was purchased from Invitrogen, and anti-CDH1 was from Santa Cruz Biotechnology.

### cDNAs

All cDNAs with/without any tag and modification (point mutations and deletions) used in this study were obtained from GeneCopoeia: FLAG-RNF157 full length, FLAG-RNF157Δ4SA (deletion of residues Ser^660–663^), FLAG-RNF157ΔRING (deletion of RING domain), FLAG-RNF157–5SA (point mutations S106A, S336A, T378A, S414A, and S572A), FLAG-RNF157-Dbox-I (point mutations R329A and L332A), FLAG-RNF157-Dbox-II (point mutations R656A and L659A), FLAG-RNF157-DDbox (double D-box mutant; R329A, L332A, R656A, and L659A), GFP-RNF157, FLAG, NIPA, FLAG-NIPA-4SA (phosphodeficient mutant), FLAG-EGFP, Myc-CDK2, Myc-CDH1, HA-ubiquitin, FLAG-EV (empty control vector), Myc-EV(empty control vector), HA-EV (empty control vector), and pReceiver-M11-FLAG-GFP.

### siRNAs

All siRNAs used in this study were purchased from Dharmacon: siRNF157-1, D-022965-01; siRNF157-2, D-022965-02; siRNF157-3, D-022965-18; siRNF157-4, D-022965–04; control siRNAs, D-001206-13.

### Other reagents

GDC-0941 (pictilisib; PI3K inhibitor) and GDC-0973 (cobimetinib; MEK inhibitor) were obtained from the Department of Chemistry at Genentech, Inc. (South San Francisco, CA). GDC-0973 and GDC-0941 drug concentrations for melanoma cell lines were selected based on conditions described previously (3), and dosing solutions were prepared in DMSO (Sigma-Aldrich). Nocodazole (M1404) and thymidine (T1895) were purchased from Sigma-Aldrich. Cell Extraction Buffer (FNN0011, Thermo Fisher Scientific), MG132 InSolution (474791, Calbiochem), CDK2 inhibitor III (Calbiochem), roscovitine (Cell Signaling Technology), and calf intestinal alkaline phosphatase (New England Biolabs) were obtained from the sources indicated. The Click-iT® EdU Assay kit was from Thermo Fisher Scientific.

### Human cell lines

Cell lines A2058, 624MEL, HeLa, and U02S were acquired from ATCC and maintained at 37 °C and 5% (v/v) CO_2_ in DMEM with 10% (v/v) FBS and 2 mm l-glutamine.

### Label-free phosphoproteomic screen

Proteins were extracted from each cell line and digested with trypsin. For phosphopeptide enrichment in serial immunoaffinity purifications, phosphomotif antibodies were used in the following order: P*X*pSP, pTP, R*X*R*XX*pS, and R*XX*pS where “*X*” represents any amino acid and “p” reflects the phosphorylation site. The samples were run in duplicate and analyzed using LTQ-Orbitrap mass spectrometry (see “Label-free phosphoproteomics”). We used a 3-fold cutoff to define regulation as 80–90% of phosphopeptide intensity changes fell within a ±3-fold change interval for most comparisons.

### Microscopy-based measurement of cell death

To quantify cell survival of A2058 and 624MEL cells after PI3K and MEK inhibition (0.625 μm), we added CellEvent® caspase-3/7 green detection reagent (Thermo Fisher Scientific) according to the manufacturer's instructions and imaged fluorescence signals at 488 nm with an IncuCyte ZOOM system (Essens Bioscience) every 90 min over 38 h at 37 °C and 5% CO_2_. Images were captured at ×20, and fluorescence signal was quantified using CellProfiler software (46).

### Stable cell line generation

cDNAs were cloned into the pShuttle-CMV/TO vector and then transferred to the Retro-GW-pHUSH-Neo retroviral destination vector by a Gateway recombination reaction (Invitrogen) as described previously (47). Retroviral vectors were co-transfected with a pVSV-G plasmid into GP2-293 packaging cells (Clontech) using FuGENE transfection reagent (Promega). Viral supernatants were collected 72 h post-transfection, centrifuged, and added to 624MEL and A2058 cells in the presence of Polybrene. Stable pools were generated by selection with Geneticin (Invitrogen).

### Affinity purification of FLAG fusion proteins

Cell lines stably expressing doxycycline-inducible FLAG fusion proteins were plated in 500-cm^2^ plates. Twenty-four hours following induction with doxycycline (1 μg/ml), cells were washed twice in ice-cold PBS and lysed in extraction buffer supplemented with a protease inhibitor mixture (Roche Applied Science), phosphatase inhibitor mixtures (Sigma), and 1 mm PMSF. After addition of extraction buffer, samples were incubated on ice for 30 min and cleared by centrifugation at 13,000 rpm for 20 min at 4 °C. Protein levels were quantified by the BCA Protein Assay kit (Pierce Biotechnology) and normalized to equal concentrations. Equal amounts of protein from each sample were precleared with mouse IgG-agarose beads (Sigma) for 2 h at 4 °C and then incubated with anti-FLAG M2 affinity agarose gel (Sigma) overnight at 4 °C. Samples were washed three times in ice-cold extraction buffer supplemented with protease and phosphatase inhibitors, resuspended in TBS-FLAG buffer (50 mm Tris-HCl, pH 7.4, 150 mm NaCl) containing 5 mg/ml 3× FLAG peptide (Sigma), and incubated for 2 h at 4 °C to elute bound protein. Eluates were concentrated by TCA precipitation and resuspended in 1× lithium dodecyl sulfate sample buffer (Invitrogen) supplemented with 1× reducing agent (Invitrogen).

### Mass spectrometry

3 × 10^8^ 624MEL and A2058 cells transfected with 500 mg of FLAG-RNF157 for 48 h were lysed in 20 mm Tris-HCl, pH 7.8, 9 mm MgCl_2_, 92 mm NaCl, 0.1% Triton X-100. FLAG-tagged RNF157 was immunoprecipitated with anti-FLAG M2 beads (Sigma) and washed in high-salt buffer (20 mm HEPES, pH 7.9, 1.5 mm MgCl_2_, 420 mm NaCl, 0.2 mm EDTA, 25% glycerol) for 10 min and then in low-salt buffer (20 mm Tris-HCl, pH 7.4, 300 mm NaCl, 0.2 mm EDTA, 20% glycerol, 0.1% Nonidet P-40) for 10 min (three repetitions) and overnight. Complexes were eluted with 500 mg/ml 3× FLAG peptide (Sigma), separated by SDS-PAGE, and then digested with trypsin. Peptides were separated by reverse-phase chromatography followed by tandem mass spectrometric analysis in an LTQ-Orbitrap (Thermo Fisher Scientific). MS/MS data were analyzed using the search algorithm Mascot (Matrix Science) and filtered to 1% peptide false discovery rate using a linear discriminant analysis as described previously (48).

### Transfection of cell lines

Transient plasmid transfections were carried out using HiPerFect transfection reagent (Qiagen), and all siRNA transfections were carried out using Lipofectamine RNAiMAX transfection reagent (Thermo Fisher Scientific) according to the manufacturers' instructions. Cells were harvested between 24 and 48 h after plasmid transfection and between 2 and 4 days for siRNA transfection.

### Cellular assays

GDC-0941 and GDC-0973 were obtained from the chemistry department at Genentech, Inc. as a 10 mm DMSO stock solution. Cell lines were maintained at 37 °C and 5% CO_2_ in RPMI 1640 medium with 10% fetal bovine serum, 2 mm l-glutamine, and penicillin-streptomycin. Cells were plated in normal growth medium at 6000 cells/well in 96-well clear-bottom black plates. The following day, cells were transfected with 100 nm siRNA using DharmaFECT 3 (Dharmacon, Chicago, IL). 48 h post-transfection, cells were treated with GDC-0973 and GDC-0941. For cell proliferation assays, 10 nm GDC-0973 and 100 nm GDC-0941 were used. For cell death assays, 20 nm GDC-0973 and 200 nm GDC-0941 were used. 24 h following drug treatment, cell proliferation was measured using the Cell Proliferation ELISA BrdU assay (Roche Applied Science), and cell death was quantified using the Cell Death Detection ELISA PLUS assay (Roche Applied Science) according to manufacturer's instructions.

### Cell treatments and cell synchronization

Cells were synchronized at G_1_/S phase by culture in 2 mm thymidine for 24 h, fresh medium for 8 h, and then 2 mm thymidine for a further 16 h. Cells were synchronized at prometaphase by culture in 2 mm thymidine for 24 h, fresh medium for 5 h, and then 100 nm nocodazole for 16 h.

### Immunoprecipitation

Cells were lysed in Cell Extraction Buffer, 1 mm PMSF (P7626, Sigma-Aldrich) protease inhibitor (11836153001, Roche Applied Science), and Phosphatase Inhibitor Mixture 2/3 (Sigma-Aldrich). Two milligrams of the lysate was incubated with 2 μg of monoclonal anti-FLAG M2 antibody (F3165, Sigma-Aldrich) or anti-Myc tag (9B11) mouse mAb (2276, Cell Signaling Technology) for 2 h at 4 °C, and immunocomplexes were precipitated using protein-G and A-Sepharose beads (17-6002-35, GE Healthcare). Beads were then washed three times with extraction buffer.

### Western blotting

To prepare protein extracts, cells were washed once with cold PBS and lysed at 4 °C in 1× Cell Extraction Buffer according to the manufacturer's instructions. For Western blot analysis, proteins were resolved by 4–12% SDS-PAGE and transferred to nitrocellulose membranes (Life Technologies). Immunoblotting was performed using the indicated primary antibodies, and immunoblots were analyzed using secondary antibodies for enhanced chemiluminescence.

### Cell cycle analyses

To analyze the cell cycle distribution, cells transfected with siRNAs were stained by using the Click-iT EdU Alexa Fluor® 647 Flow Cytometry Assay kit (Thermo Fisher Scientific) according to the manufacturer's instructions. Analyses were then performed with a FACScan cytometer (BD Biosciences) and FlowJo software.

### Label-free phosphoproteomics

For global views of phosphorylation, cell lines were probed with four phosphomotif antibodies (KinomeView®, Cell Signaling Technology) as shown in supplemental Fig. S1. For the MS-based phosphoproteomic screen, cells were washed once with cold PBS and lysed in urea lysis buffer (20 mm HEPES, pH 8.0, 9.0 m urea, 1 mm sodium orthovanadate, 2.5 mm sodium pyrophosphate, 1 mm β-glycerol phosphate). For each sample, 10 150-mm dishes of cells grown to 70–80% confluence were lysed in 10 ml of urea lysis buffer. Sonication of resulting lysates was performed at 15-watt output power twice for 20 s and once for 15 s. Sonicated lysates were centrifuged at 20,000 × *g* for 15 min to remove insoluble material, and protein extracts were reduced and carboxamidomethylated. Following normalization of total protein, proteins were digested overnight by trypsin (MAPK and tPP enrichments) or Glu-C (AKT enrichments) and subsequently trypsin-digested post-immunoaffinity purification. Resulting peptides were separated from non-peptide material by solid-phase extraction with Sep-Pak C_18_ cartridges. Lyophilized peptides were redissolved, and phosphopeptides were enriched by serial immunoaffinity purifications using slurries of the appropriate immobilized motif antibody and eluted from antibody–resin into a total volume of 100 μl in 0.15% TFA, and concentrated with C_18_ spin tips. Finally, peptides were loaded onto a 10-cm × 75-μm PicoFrit capillary column (developed with a 90-min linear gradient of acetonitrile in 0.125% formic acid delivered at 280 nl/min) packed with Magic C_18_ AQ reversed-phase resin. Using XCalibur 2.0.7 SP1, a “top 20” method, a dynamic exclusion repeat count of 1 and a repeat duration of 30 s were applied. Tandem mass spectra were collected with the LTQ-Orbitrap mass spectrometer. Real-time recalibration of mass error was applied using lock mass with a singly charged polysiloxane ion (mass-to-charge (*m*/*z*) = 371.101237). MS spectra were collected in the Orbitrap component, and MS/MS spectra were collected in the LTQ. A mass accuracy of ±50 ppm was used for precursor ions, and 1 Da was used for product ions. SEQUEST 3G and the SORCERER 2 platform from Sage-N Research (v4.0; Milpitas, CA) were used to analyze MS/MS spectra. Searches were performed against the NCBI *Homo sapiens* FASTA database updated on September 6, 2010 (release 43). A reverse decoy database was used to estimate false positive rates. Using the Peptide Prophet module of SORCERER 2, peptide assignments were obtained at a 5% false positive discovery rate. Up to four missed cleavages and peptides with one non-tryptic terminus (not cleaved after Lys or Arg) were allowed. Methionine oxidation and Ser/Thr/Tyr phosphorylation were allowed. Cysteine carboxamidomethylation was specified as a static modification. Results were further filtered for the matches with the phosphorylation motif of the respective antibodies and using mass accuracy (±5ppm) filters. The final false positive discovery rate on motif-containing peptides was lower than 1%. Changes in phosphopeptide intensities were determined by taking the ratio of averaged raw intensities between two specified conditions. We used chromatographic retention times and *m*/*z* ratios for all phosphopeptides identified in one or more samples to search for phosphopeptide ions in the ion chromatogram files. Retention time windows were variable and based on the systematic retention time deviation pattern from the extracted ion chromatograms. The *m*/*z* ranges were also variable and dependent on the mass error narrowing performed in a previous step.

### Generation of phosphospecific RNF157 antibody

The rabbits were immunized with the phosphopeptide (CRNAQRRRLpSpSpSpSLED-amide). The antibody was then purified against this phosphopeptide, which yielded two antibody populations, phosphospecific and cross-reactive antibodies. To separate these populations of antibody, the purified antibody went through the affinity absorption step against the non-phosphopeptide. This absorption step was done twice to increase the antibody specificity to the phosphopeptide.

### Statistical analysis

Data were analyzed using the unpaired *t* test with a two-tailed *p* value using a GraphPad software package (Prism 6.0). Data are expressed as mean ± S.D. A *p* value of <0.05 was considered statistically significant. *p* values are designated with asterisks as follows: * means *p* ≤ 0.05, ** means *p* ≤ 0.01, and *** means *p* ≤ 0.001.

## Author contributions

T. D., K. P. H., and G. H. conceived the project. T. D. designed and performed most biochemical and cellular experiments. M. P. S. designed the phosphoproteomic experiment. F. G. and M. P. S. analyzed the phosphoproteomic data. T. D., J. C., L. P., A. Y., and D. S. K. performed the mass spectrometry experiments. J. C. screened the top hits from the phosphoproteomic analysis by using siRNAs and performed the cell death analysis. SD. produced cell survival data by using a caspase-3/7 detection system. M. J. C. analyzed the conservation profile of RNF157. L. S. F. and M. B. participated in the design of the study and helped to revise the manuscript. T. D., F. G., and G. H. wrote the paper. All authors reviewed and approved the final version of the manuscript.

## Supplementary Material

Supplemental Data

## Footnotes

3 The abbreviations used are:

RNF157: really interesting new gene (RING) finger protein 157
CDK: cyclin-dependent kinase
APC/C: anaphase-promoting complex/cyclosome
D-box: destruction box
pRNF157: phosphorylated RNF157
APC: anaphase-promoting complex
NIPA: nuclear-interacting partner of ALK
EV: empty vector
EdU: 5-ethynyl-2′-deoxyuridine
LTQ: linear trap quadrupole
pCDK2: phosphorylated CDK2.
