# Supplementary material for: Role of the E3 ubiquitin ligase RNF157 as a novel downstream effector linking PI3K and MAPK signaling pathways to the cell cycle
Source: J Biol Chem. 2017 Jun 27;292(35):14311–24. doi: 10.1074/jbc.M117.792754 (PMC5582827; doi:10.1074/jbc.M117.792754)
Supplement: Supplemental Data [file supp_292_35_14311__index.html]

Role of the E3 ubiquitin ligase RNF157 as a novel downstream effector linking PI3K and MAPK signaling pathways to the cell cycle — Modulation of the cell cycle by RNF157 — Supplemental Data 

# Role of the E3 ubiquitin ligase RNF157 as a novel downstream effector linking PI3K and MAPK signaling pathways to the cell cycle

## Supplemental Data

- Supplemental Table 1 (.xlsx, 200 KB) - Identified phosphopeptides and their response to treatment for Figure 1
- Supplemental Table 2 (.xlsx, 189 KB) - Gene set enrichment analysis of PI3K/MEK-dependent phosphoproteins for Figure 1
- Supplemental Table 3 (.xlsx, 10 KB) - Peptide spectra matches (PSMs) demonstrating singly, doubly, triply and quadruply phosphorylated forms of RNF157 in the region covering Her residues 660-663
- Supplemental Table 4 (.xls, 28 KB) - Affinity Purification-Mass Spectrometry results from A2058 and 624MEL cell lines expressing Flag-GFP or Flag-RNF157 for putative interacting partners
- revised supplemental data (.pdf, 4.0 MB) - revised supplemental data
