# Supplementary material for: Role of the E3 ubiquitin ligase RNF157 as a novel downstream effector linking PI3K and MAPK signaling pathways to the cell cycle
Source: J Biol Chem. 2017 Jun 27;292(35):14311–24. doi: 10.1074/jbc.M117.792754 (PMC5582827; doi:10.1074/jbc.M117.792754)
Supplement: Supplemental Data [file 10.1074_M117.792754_jbc.M117.792754-5.pdf]

Supplementary Information

Role of the E3 Ubiquitin Ligase RNF157 as a Novel Downstream Effector Linking PI3K and MAPK Signaling to the Cell Cycle

Taner Dogan, Florian Gnad, Jocelyn Chan, Lilian Phu, Amy Young, Mark J. Chen, Sophia Doll, Sarah Gierke, Matthew P. Stokes, Marcia Belvin, Lori S. Friedman, Donald S. Kirkpatrick, Klaus P. Hoeflich and Georgia Hatzivassiliou

Figure S1. Inactivation of PI3K/MAPK by inhibitors triggers cell death

Figure S2. Identification of RNF157 as a novel PI3K-MAPK pathway node by phosphoproteomic analysis

Figure S3. The conservation profile of RNF157

Figure S4. Analysis of RNF157 wildtype and D-box domain mutant expression and Cdh1 interaction

Figure S5. Current working model for RNF157 regulation by Cdh1 binding and CDK2 phosphorylation, downstream of PI3K/MEK activation during the cell cycle

Table S1. Identified phosphopeptides and their response to treatment for Fig 1

Table S2. Gene set enrichment analysis of PI3K/MEK-dependent phosphoproteins for Fig 1

Table S3. Peptide spectra matches (PSMs) demonstrating singly, doubly, triply and quadruply phosphorylated forms of RNF157 in the region covering Ser residues 660-663

Table S4. Affinity Purification-Mass Spectrometry results from A2058 and 624-Mel cell lines expressing Flag-GFP or Flag-RNF157 for putative interacting partners

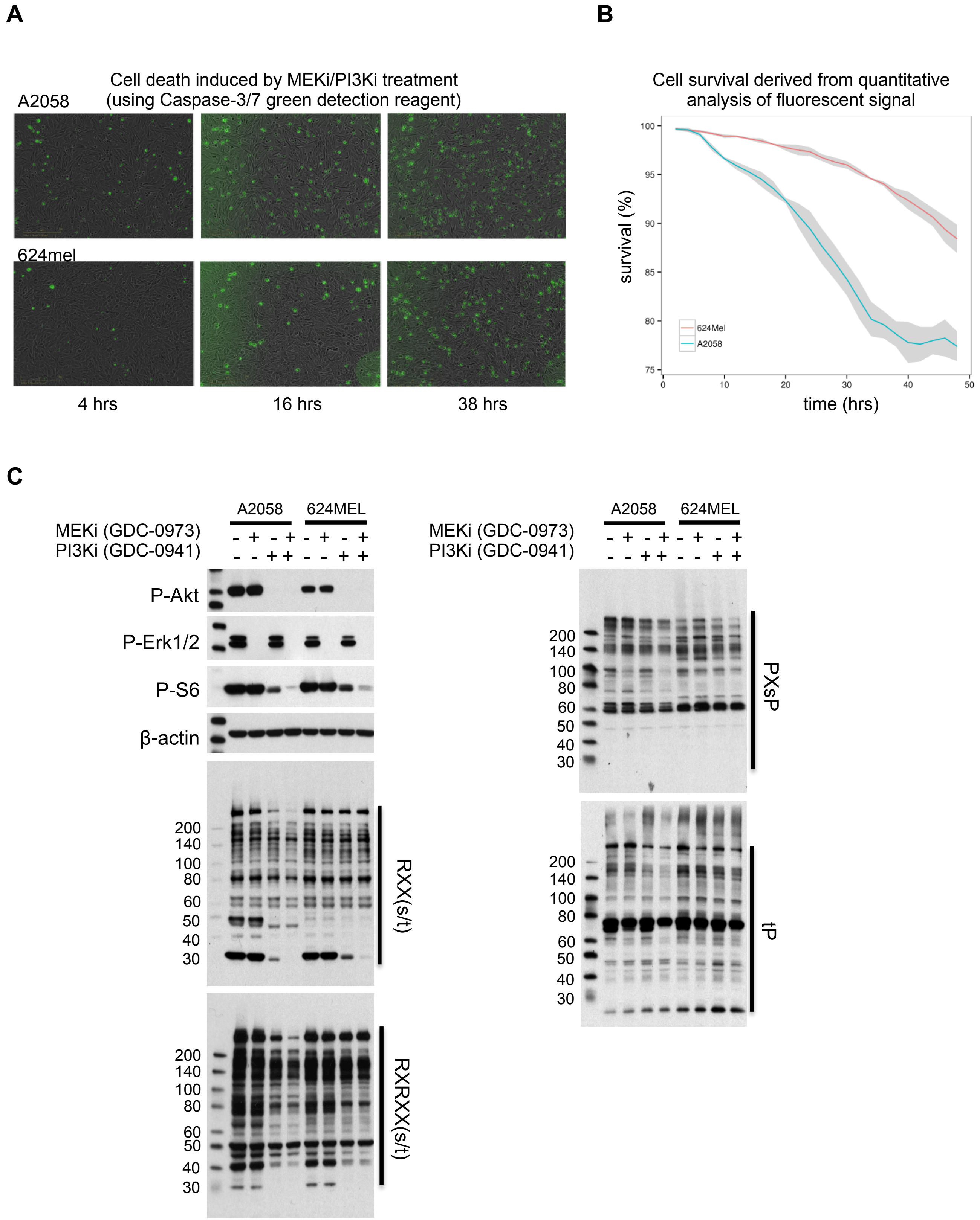

Figure S1. Inactivation of PI3K/MAPK by inhibitors triggers cell death. (A) Illustration of PI3K/MAPK inhibition (0.5 μM) induced cell death after 16h by green fluorescent signal of caspase 3/7 detection reagent. (B) Cell survival derived from quantitative analysis of fluorescent signal in A2058 and 624MEL cells after PI3K/MAPK inhibition. (C) Western blots illustrate phosphorylation changes of PI3K and MAPK activity markers after PIK3CA and/or MEK inhibition (0.5 μM each for 2 hr). In addition, global phosphorylation profiling was performed using phosphomotif antibodies.

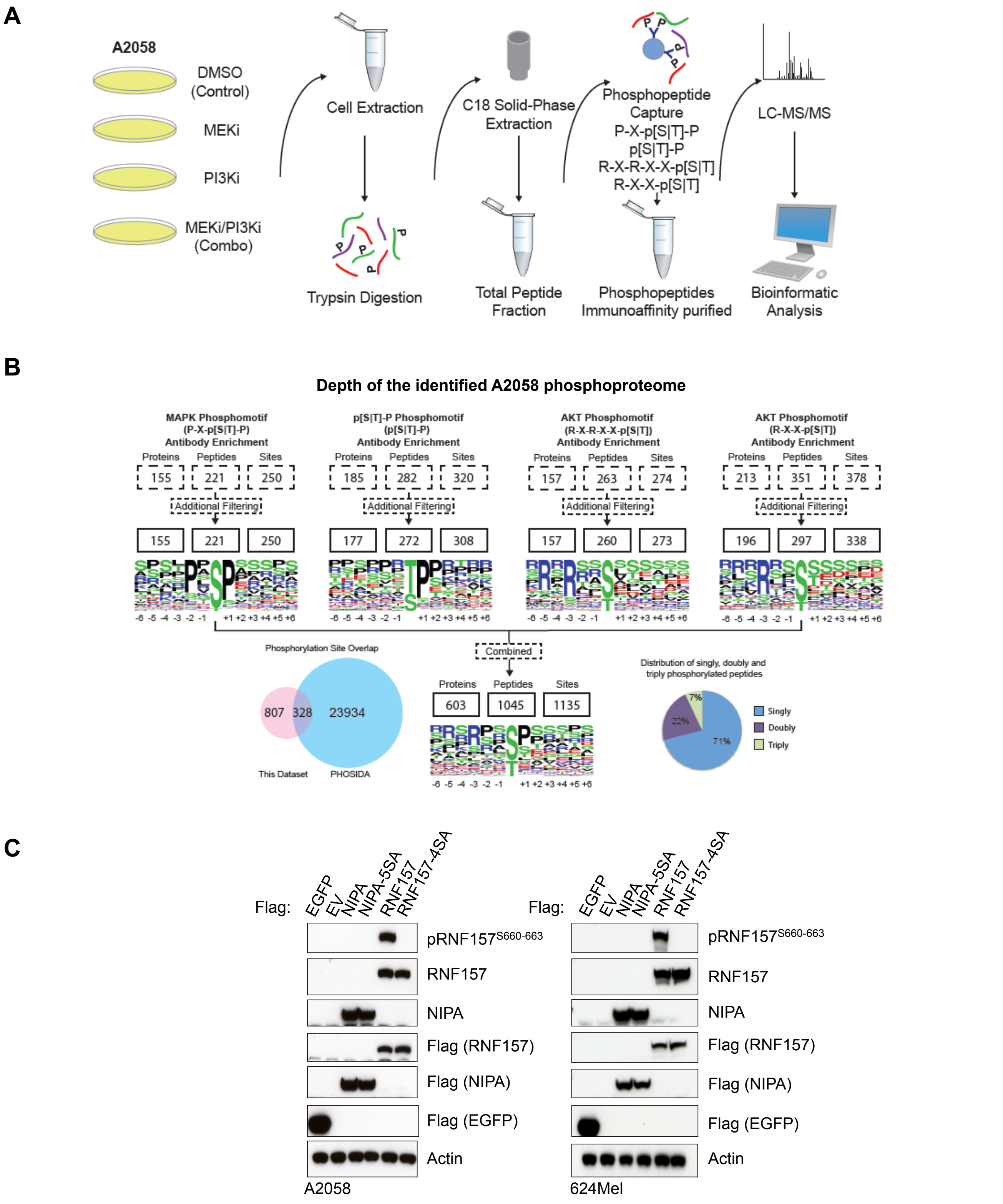

Figure S2. Identification of RNF157 as a novel PI3K-MAPK pathway node by phosphoproteomic analysis. (A) Illustration of the mass spectrometry-based label-free approach (explained in the section of Materials and Methods). (B) Boxes show the number of identified phospho-proteins, -peptides, and –sites. Sequence logos display the relative frequency of amino acids in proximity to identified phosphosites. Pie charts illustrate overlap with the Post-Translation Modification Database PHOSIDA (www.phosida.com) and the number of singly- and multiply-phosphorylated peptides. (C) Validation of the phospho-specific RNF157 antibody, pRNF157-S660-663. A2058 and 624Mel cells were transfected with Flag-EGFP, Flag-EV (empty vector), Flag-NIPA, Flag-phospo-deficient NIPA mutant (NIPA-5SA), Flag-RNF157 or Flag-phospho-deficient RNF157 mutant (RNF157-4SA) expression vector separately. Lysates were blotted with anti-phospho-RNF157S660-663, RNF157, Flag, NIPA, Actin antibodies.

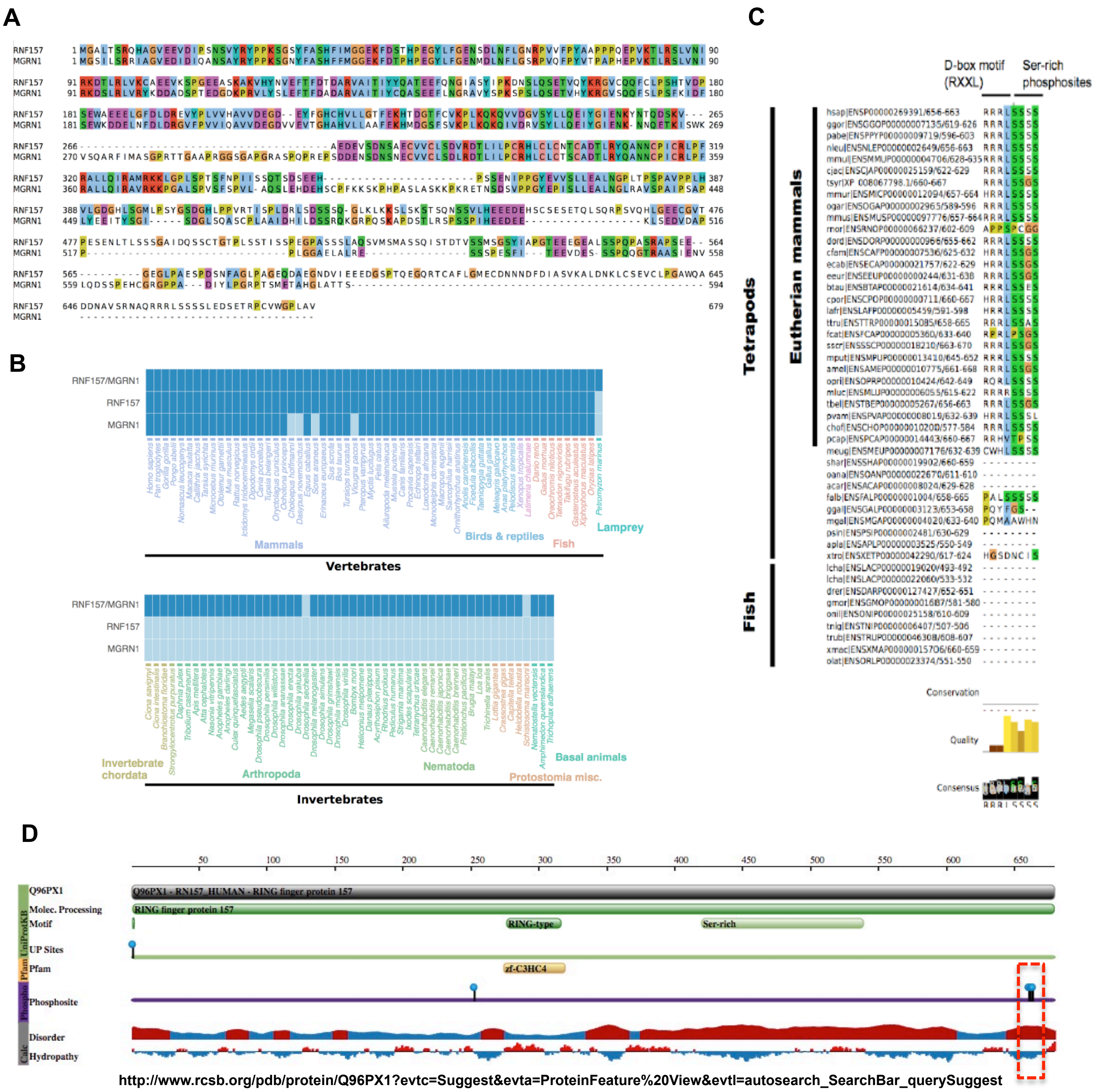

Figure S3. The conservation profile of RNF157. (A) Needleman-Wunsch alignment between human RNF157 and MGRN1 using the EMBOSS package (1). JalView (2) was used to visualize the alignment. (B) Phylogenetic profile of RNF157 and MGRN1. RNF157 and MGRN1 orthologs are found in almost all jawed vertebrates (the dropouts are in incompletely requences genomes). A single RNF157/MGRN1 co-ortholog is found in all major eukaryotic lineages other than most fungi. Using BLAST (3) and MCL (4) we derived the orthologs of RNF157 and MGRN1 across 203 species. The proteomes from 203 species were retrieved from Ensembl (http://ensembl.org) or JGI (http://genome.jgi.doe.gov). (C) Multiple sequence alignment of RNF157 orthologs shows that the C-terminal D-box motif and Ser 660-663 is conserved in eutherian mammals but degenerate or absent from non-mammalian vertebrates. We aligned the sequence using Clustal Omega (5) followed by manual adjustment. The conservation score is shown in the lower panel. JalView was used to visualize the alignment. (D) Protein Feature View of RNF157 provides a graphical summary of full-length RNF157 relative to PDB entries using annotations from external databases such as Pfam and Phosphosite and predicted regions of protein disorder (computed with JRONN) and hydrophobic regions, computed using a sliding window approach. Predicted disordered and hydrophobic regions are in red; ordered and hydrophilic regions are in blue. According to this analysis, the S660-663 region lies within a predicted disordered region. Protein disorder predictions are based on JRONN (Troshin, P. and Barton, G. J. unpublished), a Java implementation of RONN (6).

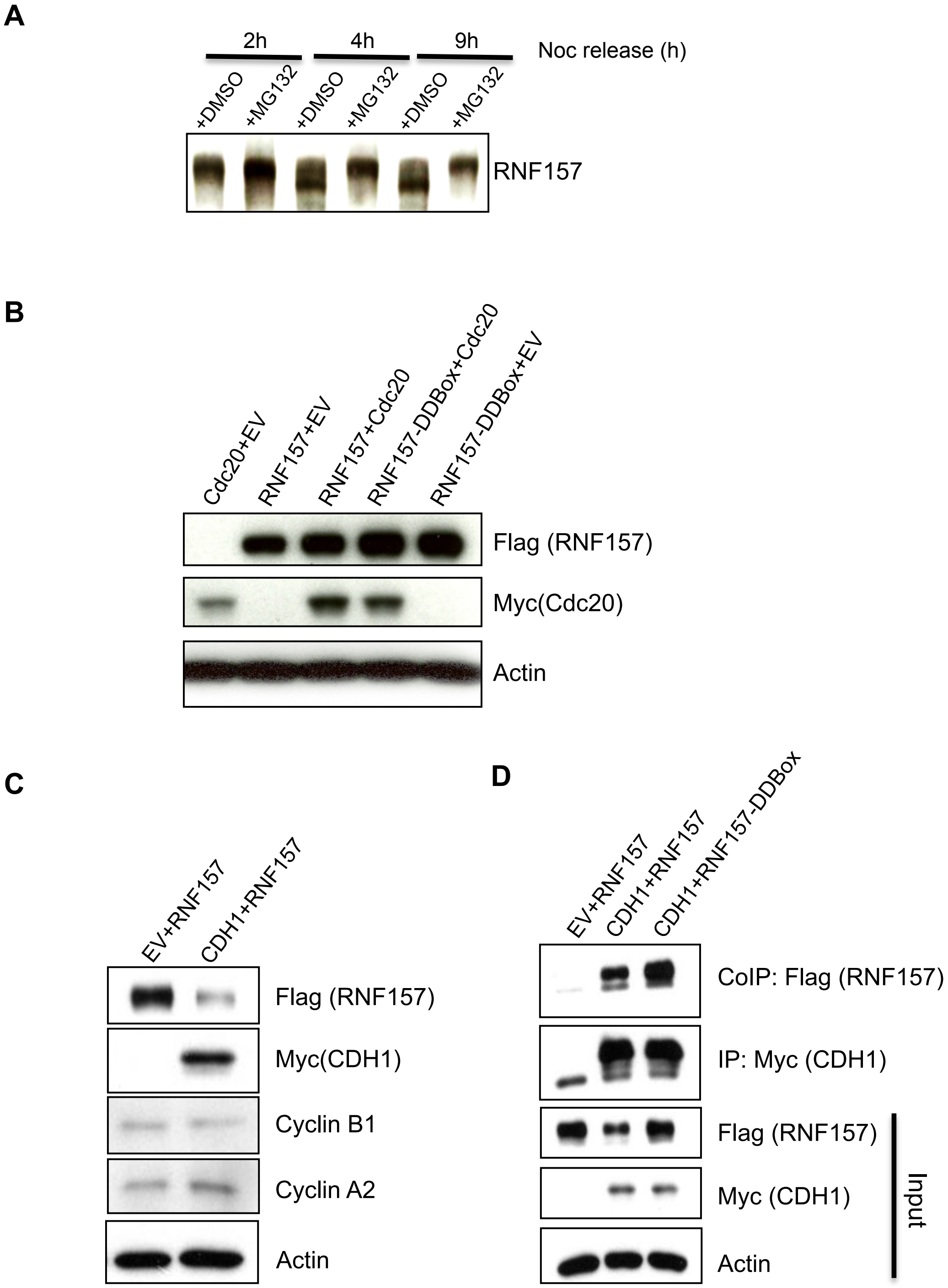

Figure S4. Analysis of RNF157 wildtype and D-box domain mutant expression and Cdh1 interaction. (A) A2058 melanoma cells were treated with nocodazole for 18 h and then released into fresh medium with/without MG132 for the times indicated. The cell lysates then were analyzed by western blotting for endogenous RNF157. (B) Lysates of 624MEL melanoma cells transfected with Flag-tagged RNF157 (RNF157) or Flag-tagged double D-box deficient mutant (RNF157-DDBox) together with control vector (EV) or Myc-Cdc20 (Cdc20). Blots were probed against Flag, Myc and actin antibodies as indicated. (C) A2058 cells were transfected with wild type Flag-RNF157 (RNF157) together with control vector (EV, empty vector) or Myc-Cdh1. Cells were lysed 48 h post transfection and lysates were blotted with anti-Flag (RNF157), anti-Myc (Cdh1), Cyclin B1, Cyclin A2 and Actin antibodies, the latter as loading control. (D) A2058 cells were transfected with wild type Flag-RNF157 (RNF157) or double D-Box mutant Flag-RNF157 (RNF157-DDbox) together with control vector (EV, empty vector) or Myc-Cdh1. Cells were lysed 48 h post transfection and lysates were immunoprecipitated with Myc antibody, followed by western blotting using Flag and Myc antibodies (top). Total lysates were also blotted with Flag, Myc and Actin to probe input protein levels (bottom).

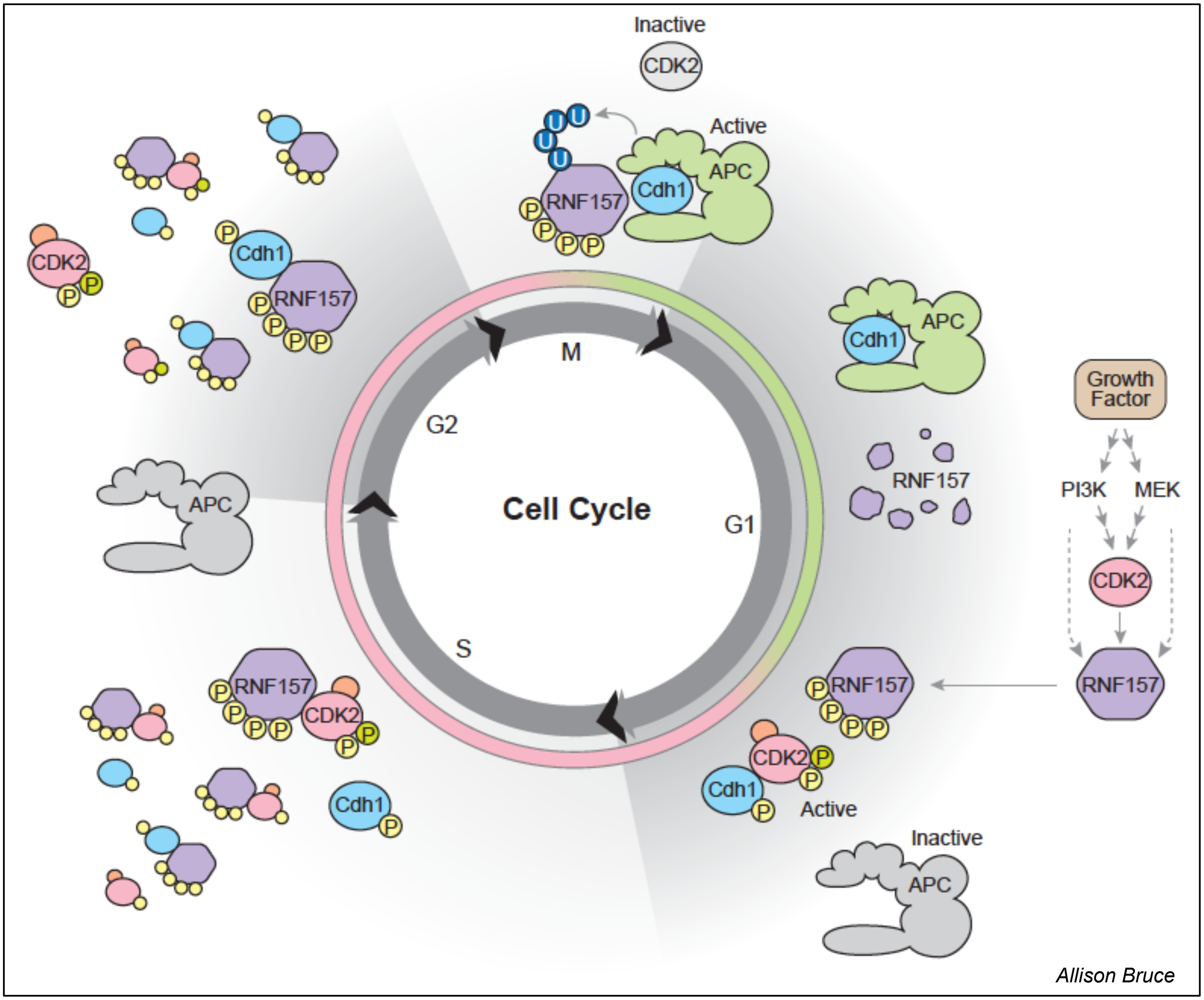

Figure S5. Current working model for RNF157 regulation by Cdh1 binding and CDK2 phosphorylation, downstream of PI3K/MEK activation during cell cycle progression. Schematic of protein:protein interactions and phosphorylation as a function of cell cycle phases, consistent with data presented in the manuscript and prior cited work regarding PI3K/MEK, CDK2 and APC/C-Cdh1 crosstalk within the cell cycle. According to this model, starting with the G1/S transition bottom right, RNF157 becomes phosphorylated on S660-663 cluster (yellow P) upon CDK2 activation downstream of PI3K/MEK activity during G1/S transition. CDK2 is itself activated by phosphorylation and in addition to RNF157 also phosphorylates Cdh1, dissociating it from the APC/C, which leads to the inactivation of APC/C-Cdh1 until late M, when CDK2 activity wanes. (Note: the pink-colored segment of the outer circle around the cell cycle phases in the diagram represents active CDK2, while the green-colored segment represents active APC/C-Cdh1) During S phase, pRNF157S660-663 interacts with CDK2, in a manner that may be promoted by its C-terminal Cy motif and remains stable and able to perform its role. Its interaction with Cdh1 gradually increases in a S660-663-dependent manner, with high RNF157/Cdh1 binding during G2/M. This allows for rapid RNF157 degradation during late M phase, as soon as the APC/C-Cdh1 complex becomes active and tight RNF157 modulation in synchrony with the cell cycle. RNF157 levels remain low during G1, since APC/C-Cdh1 is still active, but begin increasing again at the G1/S checkpoint, upon growth factor and PI3K/MEK pathway stimulation and CDK2 activation.

Ascore Sequence	PPM	Mascot Ion Score	Ascore 1	Ascore 2	Ascore 3	Ascore 4	Sequence motif	# phos
RRLS#S#S#S#LEDSETRPCVWGPLAV	6.38	28.76	68	61.7	54.7	59.1	RRLS#S#S#S#LED	4
RL[SSS]##S#LEDSETRPCVWGPLAV	5.48	36.12	11.5	7.8	28.2	0	RRL[SSS]##S#LED	3
RRLS#[SSS]#LEDSETRPCVWGPLAV	5.24	52.45	20.4	8.7	0	0	RRLS#[SSS]#LED	2
RLSSS#S#LEDSETRPCVWGPLAV	5.27	47.63	19.2	36.7	0	0	RRLSSS#S#LED	2
RLS#S#SSLEDSETRPCVWGPLAV	6.2	69.29	14.4	14.4	0	0	RRLS#S#SSLED	2
RRL[SSS]#S#LEDSETRPCVWGPLAV	5.91	26.1	12.2	38.4	0	0	RRL[SSS]#S#LED	2
RLS#SSSLEDSETRPCVWGPLAV	4.69	55.4	19.3	0	0	0	RRLS#SSSLED	1
RLSS#SSLEDSETRPCVWGPLAV	4.38	53.45	19.2	0	0	0	RRLSS#SSLED	1

Table S3. Peptide spectra matches (PSMs) demonstrating singly, doubly, triply and quadruply phosphorylated forms of RNF157 in the region covering Ser residues 660-663. Precursor ion mass accuracy and Mascot ion scores are reported for each PSM. Ascore values represent the confidence of site localization, with scores of 13 and 19 representing 95% and 99% confidence in the site assignment. Square brackets denote regions of ambiguity for sequence localization of confidently identified phosphorylation sites. Singly phosphorylated peptides with confidently assigned sites (>99%) confirm mono-phosphorylation at Ser660 and Ser661. A quadruply phosphorylated peptide with concurrent phosphorylation at each of the Ser residues between 660-663. Permutations of doubly and triply phosphorylated peptides covering this same sequence were also confidently identified

Reference	Description	A2058 Flag-GFP	A2058 Flag-RNF	624MEL Flag-GFP	624MEL Flag-RNF	Total
ATRN_HUMAN	Attractin	0 (0)	40 (20)	0 (0)	32 (16)	72
MEGF8_HUMAN	Multiple epidermal growth factor-like domains protein 8	0 (0)	26 (22)	0 (0)	10 (10)	36
TECR_HUMAN	Trans-2,3-enoyl-CoA reductase	0 (0)	9 (8)	0 (0)	19 (12)	28
MSI2H_HUMAN	RNA-binding protein Musashi homolog 2	0 (0)	10 (5)	0 (0)	17 (5)	27
PLRG1_HUMAN	Pleiotropic regulator 1	0 (0)	10 (8)	0 (0)	11 (10)	21
BYST_HUMAN	Bystin	0 (0)	8 (8)	1 (1)	10 (10)	18
MTER1_HUMAN	mTERF domain-containing protein 1, mitochondrial	0 (0)	11 (10)	0 (0)	6 (5)	17
PSA1_HUMAN	Proteasome subunit alpha type-1	0 (0)	10 (8)	0 (0)	7 (5)	17
RT18B_HUMAN	28S ribosomal protein S18b, mitochondrial	0 (0)	9 (8)	0 (0)	8 (8)	17
PRP4_HUMAN	U4/U6 small nuclear ribonucleoprotein Prp4	0 (0)	8 (8)	1 (1)	8 (8)	16
FAKD2_HUMAN	FAST kinase domain-containing protein 2	0 (0)	6 (5)	0 (0)	9 (8)	15
TXTP_HUMAN	Tricarboxylate transport protein, mitochondrial	0 (0)	8 (6)	0 (0)	7 (6)	15
SMU1_HUMAN	WD40 repeat-containing protein SMU1	0 (0)	5 (5)	0 (0)	10 (9)	15
RCD1_HUMAN	Cell differentiation protein RCD1 homolog	1 (1)	5 (5)	0 (0)	10 (10)	15
RT02_HUMAN	28S ribosomal protein S2, mitochondrial	0 (0)	6 (5)	0 (0)	9 (8)	15
MAGT1_HUMAN	Magnesium transporter protein 1	0 (0)	7 (6)	0 (0)	8 (7)	15
CHD1_HUMAN	Chromodomain-helicase-DNA-binding protein 1	0 (0)	8 (8)	0 (0)	7 (7)	15
FXR2_HUMAN	Fragile X mental retardation syndrome-related protein 2	1 (1)	7 (7)	0 (0)	7 (6)	14
EMD_HUMAN	Emerin	0 (0)	6 (5)	0 (0)	8 (8)	14
PSMD8_HUMAN	26S proteasome non-ATPase regulatory subunit 8	0 (0)	7 (6)	0 (0)	6 (6)	13
HDAC1_HUMAN	Histone deacetylase 1	0 (0)	7 (7)	0 (0)	6 (5)	13
RAN_HUMAN	GTP-binding nuclear protein Ran	0 (0)	6 (5)	0 (0)	6 (5)	12
DHB12_HUMAN	Estradiol 17-beta-dehydrogenase 12	0 (0)	5 (5)	0 (0)	7 (7)	12
TXND5_HUMAN	Thioredoxin domain-containing protein 5	1 (1)	7 (7)	0 (0)	5 (5)	12
RM19_HUMAN	39S ribosomal protein L19, mitochondrial	0 (0)	5 (5)	0 (0)	6 (5)	11

Table S4. Affinity Purification-Mass Spectrometry results from A2058 and 624MEL cell lines expressing Flag-GFP or Flag-RNF157. Values in each column represent the Total (Unique) number of peptide spectral matches (PSMs) corresponding to each Reference protein. Proteins with less than 2 PSMs in both GFP control cell lines and at least 5 Unique PSMs in at least one of the RNF157 expressing samples are displayed.

Supplemental References

1. Rice, P., Longden, I., and Bleasby, A. (2000) EMBOSS: the European Molecular Biology Open Software Suite. *Trends Genet* **16**, 276-277
2. Waterhouse, A. M., Procter, J. B., Martin, D. M., Clamp, M., and Barton, G. J. (2009) Jalview Version 2--a multiple sequence alignment editor and analysis workbench. *Bioinformatics* **25**, 1189-1191
3. Altschul, S. F., Madden, T. L., Schaffer, A. A., Zhang, J., Zhang, Z., Miller, W., and Lipman, D. J. (1997) Gapped BLAST and PSI-BLAST: a new generation of protein database search programs. *Nucleic Acids Res* **25**, 3389-3402
4. Enright, A. J., Van Dongen, S., and Ouzounis, C. A. (2002) An efficient algorithm for large-scale detection of protein families. *Nucleic Acids Res* **30**, 1575-1584
5. Sievers, F., Wilm, A., Dineen, D., Gibson, T. J., Karplus, K., Li, W., Lopez, R., McWilliam, H., Remmert, M., Soding, J., Thompson, J. D., and Higgins, D. G. (2011) Fast, scalable generation of high-quality protein multiple sequence alignments using Clustal Omega. *Mol Syst Biol* **7**, 539
6. Yang, Z.R., Thomson, R., McNeil, P., and Esnouf, R.M. (2005) RONN: the bio-basis function neural network technique applied to the detection of natively disordered regions in proteins. *Bioinformatics* **21**, 3369
